# Radio-enhancement effects by radiolabeled nanoparticles

**DOI:** 10.1038/s41598-019-50861-2

**Published:** 2019-10-04

**Authors:** Yaser Hadi Gholami, Richard Maschmeyer, Zdenka Kuncic

**Affiliations:** 1 The University of Sydney, Institute of Medical Physics, School of Physics, Sydney, NSW 2006 Australia; 2 The University of Sydney Nano Institute, Sydney, NSW 2006 Australia

**Keywords:** Nanotechnology in cancer, Biological physics

## Abstract

In cancer radiation therapy, dose enhancement by nanoparticles has to date been investigated only for external beam radiotherapy (EBRT). Here, we report on an *in silico* study of nanoparticle-enhanced radiation damage in the context of internal radionuclide therapy. We demonstrate the proof-of-principle that clinically relevant radiotherapeutic isotopes (i.e. ^213^Bi, ^223^Ra, ^90^Y, ^177^Lu, ^67^Cu, ^64^Cu and ^89^Zr) labeled to clinically relevant superparamagnetic iron oxide nanoparticles results in enhanced radiation damage effects localized to sub-micron scales. We find that radiation dose can be enhanced by up to 20%, vastly outperforming nanoparticle dose enhancement in conventional EBRT. Our results demonstrate that in addition to the favorable spectral characteristics of the isotopes and their proximity to the nanoparticles, clustering of the nanoparticles results in a nonlinear collective effect that amplifies nanoscale radiation damage effects by electron-mediated inter-nanoparticle interactions. In this way, optimal radio-enhancement is achieved when the inter-nanoparticle distance is less than the mean range of the secondary electrons. For the radioisotopes studied here, this corresponds to inter-nanoparticle distances <50 nm, with the strongest effects within 20 nm. The results of this study suggest that radiolabeled nanoparticles offer a novel and potentially highly effective platform for developing next-generation theranostic strategies for cancer medicine.

## Introduction

Radiation is used in approximately 50% of all cancer treatments^1^. The key objective in cancer radiotherapy is to achieve a high therapeutic efficacy by maximizing damage to the tumor whilst minimizing damage to surrounding healthy tissue^2–4^. In conventional external beam radiotherapy, the dose that can be delivered to a tumor is often limited by the presence of an adjacent critical organ. This means that not all tumor cells may receive a lethal dose of radiation, thus limiting the treatment efficacy. Radiotherapy is constantly being transformed by new technologies and one of the most promising developments is the use of high atomic number (high-*Z*) nanoparticles to locally enhance radiation-induced tumor cell kill^5–7^. In nanoparticle radio-enhancement strategies, the increased probability of radiation interactions in the presence of high-*Z* nanoparticles results in the release of copious numbers of secondary particles (mostly low-energy electrons) that can enhance local radiation damage effects^8–10^, thus increasing the probability of tumor cell kill without affecting surrounding healthy tissue^11–14^.

The effects of radio-enhancement and its dependence on cluster morphology have been investigated in many *in vivo*, *in vitro* and *in silico* studies^5,15–20^. To date, however, these studies have almost exclusively considered radiation delivered by an external beam (i.e. external beam radiotherapy). Targeted internal radionuclide therapy is an alternative treatment approach to achieve more localized radiotherapy by delivering a radioisotope internally to a tumor^21,22^. Nanoparticles can be labeled with various radioisotopes for use in both internal radionuclide therapy and diagnostic imaging (emission tomography)^23^. Only one previous *in silico* study, by Sung *et al*.^24^, considered nanoparticle dose enhancement for the radioisotope sources ^111^In and ^99m^Tc used in Auger therapy. However, the study only simulated a single nanoparticle. Here, nanoparticle-enhanced radiation damage in the context of internal radionuclide therapy is demonstrated using computational modelling to simulate the more realistic scenario of clusters of nanoparticles and to explore radio-enhancement effects for a range of clinically used radioisotopes. Nanoparticles containing a superparamagnetic iron oxide (SPIO) core were chosen for this study because of the relatively high atomic number of iron (i.e. *Z* = 26), their excellent biocompatibility, and because they also enhance contrast in magnetic resonance imaging, making them ideal theranostic candidates^25–29^.

Therapeutic radioisotopes emit three main types of radiation for internal radionuclide therapy: γ particles (photons), *β*^−^ particles (electrons), *α* particles (helium nuclei), and Auger electrons^30–35^. As the energy of the emitted radiation particles is typically in the kilo-electronvolt (keV) range, the probability of interaction with high-*Z* nanoparticles can be significantly higher than that for a conventional external radiation beam (photon or charged particle), which is typically in the mega-electronvolt (MeV) energy range^36^. For photons, the photoelectric effect dominates at keV energies and has a sensitive dependence on Z. In water, the inelastic mean free path for sub-keV (i.e. <100 eV) charged particles increases significantly with decreasing charged particle energy, while for higher energy charged particles, the inelastic mean free path increases with energy^37^, although the dependence on Z is less sensitive than for photo-ionization, so radio-enhancement relies solely on the high density of nanoparticles. In the context of radiolabeled nanoparticles considered here, the overall interaction probability is increased by the close proximity of the radiation source to the nanoparticles. Thus, radio-enhancement by nanoparticles should be more significant for internal radionuclide therapy than for external beam radiotherapy. This proof-of-principle is demonstrated for the first time in the present study.

## Results

Previous studies have shown that the FDA approved nanoparticle Feraheme® (FH) can be radiolabeled with isotopes using a novel chelate-free technique^23^ in which the radioisotopes bind directly to the surface of the SPIO core. Hence, direct interaction of emitted radiation particles with the SPIO core can potentially result in enhancing local energy deposition. Here, nanoparticle-enhanced radiation damage in the context of internal radionuclide therapy is demonstrated using computational modelling to simulate all possible interactions and calculate radiation damage effects in terms of relevant quantities such as dose, particle hits and secondary particle production.

### Two-dimensional (2D) histograms for dose and particle hits

**Figure 1:**
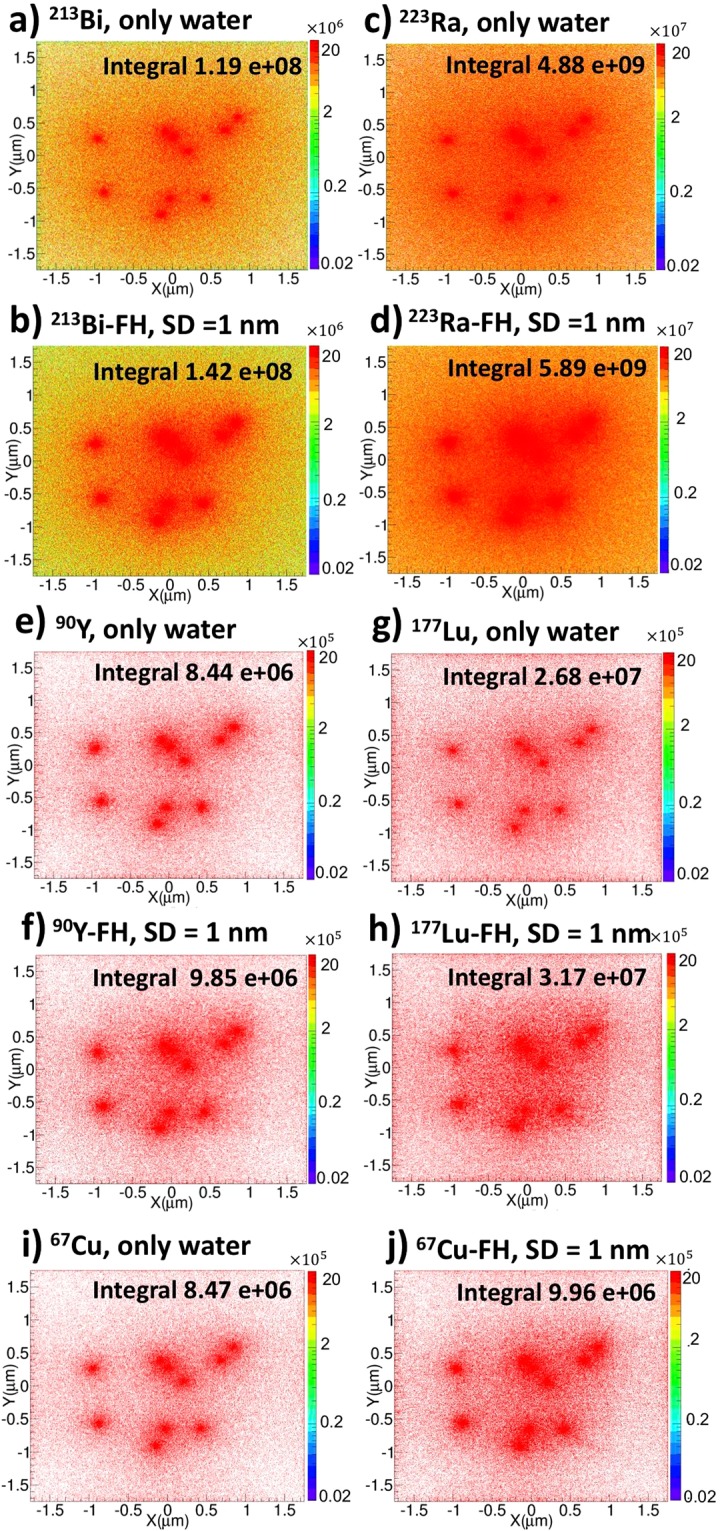
Two-dimensional (2D) dose histograms for therapeutic radioisotopes with and without Feraheme (FH) nanoparticles. The colourbar is in Gy and the total integrated dose in Gy is indicated; (**a**) and (**b**) ^213^Bi only and ^213^Bi-FH, respectively; (**c**) and (**d**) ^223^Ra only and ^223^Ra-FH, respectively; (**e**) and (**f**) ^90^Y only and ^90^Y-FH, respectively; (**g**) and (**h**) ^177^Lu only and ^177^Lu-FH, respectively; (**i)** and (**j**) ^67^Cu only and ^67^Cu-FH, respectively.

**Figure 2:**
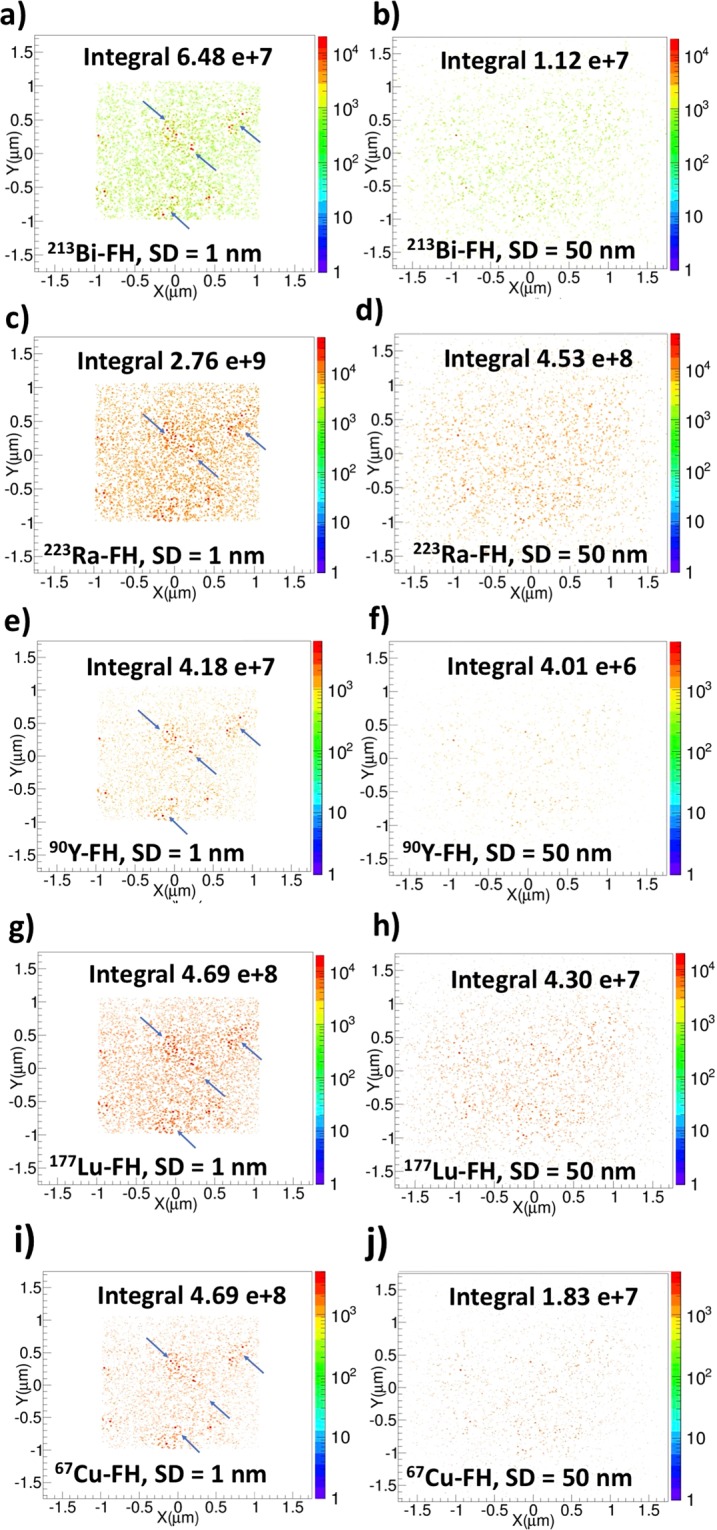
Two-dimensional particle hits distributions resulting from radiolabeled Feraheme (FH) for nanoparticle separation distances (SD) up to 1 nm and up to 50 nm. Blue arrows point to regions with locally-enhanced particle hits. The total integrated number of hits is indicated.

Figure 1(a–j) shows 2D histograms of dose by integrating the corresponding 3D dose distribution along the *Z* direction. For each case, the spatial distribution of dose exhibits a qualitative difference when the isotope sources are uniformly distributed throughout a water phantom without NPs compared to when NPs are present, with the latter case resulting in a noticeable increase in intensity around the immediate vicinity of radiolabeled FH (radio-FH). Quantitatively, the total dose is higher when the NPs are present, with the largest increase of 21% found for ^223^Ra-FH. This suggests that at smaller separation distances (SDs), the nanoparticle clustering results in a collective effect that enhances the dose by electron-mediated inter-nanoparticle interactions. Similarly, a previous study^20^ also found that when a cluster (with separation distance, SD 1 nm) of gold nanoparticles (GNPs, with r = 50 nm) randomly distributed in a water phantom is irradiated with a keV external photon beam, the secondary electrons produced from neighboring GNPs contribute to local dose in the periphery of a GNP and thus enhance dose. On the other hand, another study^38^ found that for closely packed GNPs (with r = 25 nm) in a three-dimensional hexagonal arrangement, the clustering mitigates dose enhancement due to the self- absorption by the GNPs for both keV and MeV external photon beam irradiation. These results suggest in both RNT and external beam radiotherapy, the clustering effect on dose enhancement depends on the size and density of the nanoparticles as well as the cluster geometry. Self-absorption effects may possibly also play a role, however better cross-sections models are needed for high-Z materials^39^. Figure 2(a–j) shows 2D particle hits distributions for radio-FH within the NP clusters for two separation distances (SDs), up to 1 nm and 50 nm. ^223^Ra-FH and ^213^Bi-FH show the highest total number of hits at both SDs, while ^90^Y-FH shows the lowest. These results also demonstrate that at separation distance (SD) up to 1 nm, NPs in close proximity to radio-FH receive on average a higher number of hits (these regions are shown by blue arrows in Fig. 2) and as SD increases, the number hits to all nanoparticles reduces and becomes more uniform. This also suggests that dose enhancement at larger SDs is mainly due to the interaction of the primary particles with the individual high-Z SPIO core. This is consistent with a previous study^40^ in which a fivefold increase was found in the dose-enhancing effect by irradiating an individual Fe_3_O_4_ nanoparticle with 70 and 150 MeV proton beams (i.e. densely ionizing charged particles with similar radiation characteristics to the radioisotopes used in this study).

To further investigate the statistical uncertainties associated with the nanoparticle spatial distributions, additional simulations (N = 2) were performed with different random number seeds to generate different statistical realizations (i.e. randomly sampled spatial coordinates). These simulations were performed for the ^223^Ra-FH with SD = 10 nm case and the statistical variance was found to be very small (i.e. mean dose = 4.8803 × 10^9^ with stdev ≈0.01%, N = 3), certainly smaller than other potential sources of systematic uncertainties in our study.

### Energy spectrum of secondary particles

**Figure 3:**
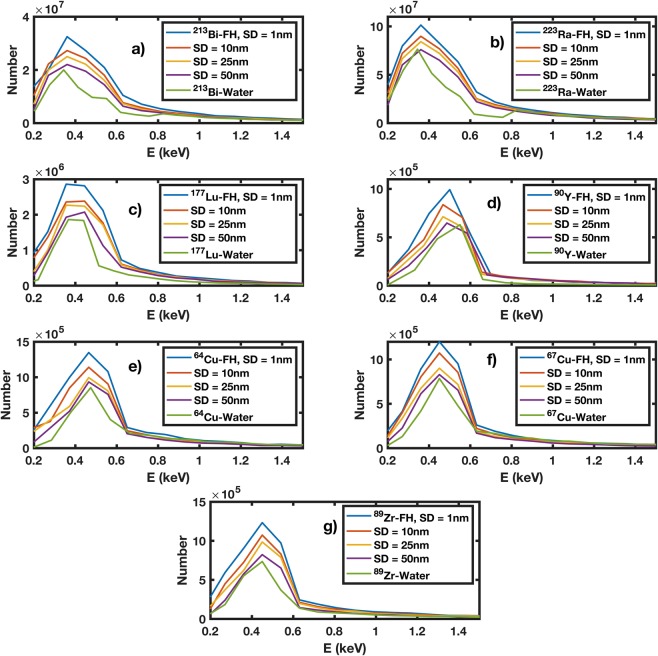
Spectrum of secondary particles (i.e. *e*^−^) resulting from radiolabeled Feraheme (FH) for varying nanoparticle separation distances (SD): (**a**) ^213^Bi-FH; (**b**) ^223^Ra-FH; (**c**) ^177^Lu-FH; (**d**) ^90^Y-FH; (**e**) ^64^Cu-FH; (**f**) ^67^Cu-FH; and (**g**) ^89^Zr-FH. Also shown are the spectra resulting from the radioisotopes in water without NPs.

Figure 3(a–g) shows the energy spectrum of secondary particles (i.e. *e*^−^) for all therapeutic and imaging radio-FH NPs for different SDs (up to 1, 10, 25 and 50 nm) compared against isotopes in water without NPs. The energy of secondary particles include the first-collision spectrum, delta and Auger electrons recorded in the space between the nanoparticles only. In all cases, the number of secondary electrons is reduced by increasing the maximum SD and the energy spectra converge to that for isotopes in water at SD = 50 nm. The maximum increase in number of secondary particles is ≈25% for ^223^Ra-FH with maximum SD = 1 nm. The number of secondary electrons generated by the radio-FH NPs peaks at ≈ 400–500 eV, corresponding to a range in water of ≈12–18 nm. The number of secondary electrons generated by the alpha emitting radio-FH NPs (i.e. ^223^Ra-FH and ^213^Bi-FH) peaks at ≈300–500 eV for SD = 1 nm, corresponding to a range in water of ≈11–14 nm. In comparison, ^223^Ra-Water and ^213^Bi-Water show slightly narrower peaks at ≈300–400 eV corresponding to a range in water of ≈11–14 nm. Additionally, the spectra intensity for all radio-FH NPs at SD = 1 nm increases by ≈30% compared to all isotope-Water cases. This suggests that NP radio-enhancement effects should be strongest for SD < 20 nm, where electron-mediated inter-nanoparticle interactions are maximized.

### Auger electron production and dose enhancement

**Figure 4:**
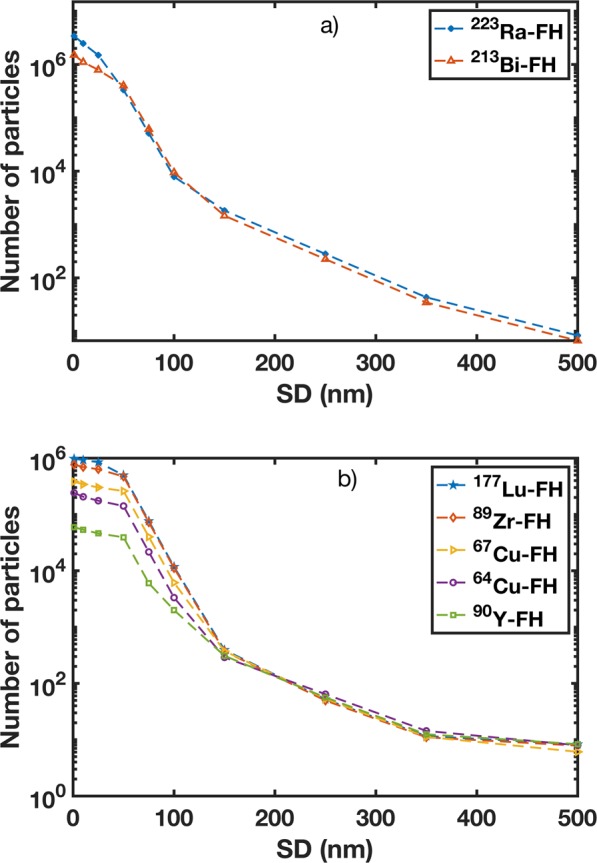
Number of Auger electrons emitted from therapeutic and imaging radioisotopes labeled to Feraheme (FH) for varying nanoparticle separation distances (SDs) for: (**a**) ^223^Ra-FH and ^213^Bi-FH; and (**b**) ^177^Lu-FH, ^90^Y-FH, ^64^Cu-FH, ^67^Cu-FH, and ^89^Zr-FH.

Figure 4(a,b) shows the number of Auger electrons produced as a function of maximum NP SD. ^213^Bi-FH and ^223^Ra-FH produce the highest number of Auger electrons at all SDs. For SD up to 1 nm, the number of Auger electrons produced is increased by 96% compared to SD up to 500 nm. Additionally, although the number of Auger electrons decreases at SD >50 nm for all radio-FH sources, the number generated by ^213^Bi-FH and ^223^Ra-FH remains above that of the number generated by other radio-FH sources. This is mainly due to the higher linear energy transfer (LET) of alpha particles (compared to *β*^−^ particles) and the stochastic nature of their energy deposition^21^. The number of Auger electrons produced in water only (i.e. without NPs) was 4.1 × 10^5^, 1.1 × 10^5^, 2.8 × 10^4^, 7.7 × 10^4^, 3.6 × 10^4^, 1.6 × 10^4^, for ^223^Ra, ^213^Bi, ^177^Lu, ^89^Zr, ^67^Cu, ^64^Cu and ^90^Y isotopes, respectively.

**Figure 5:**
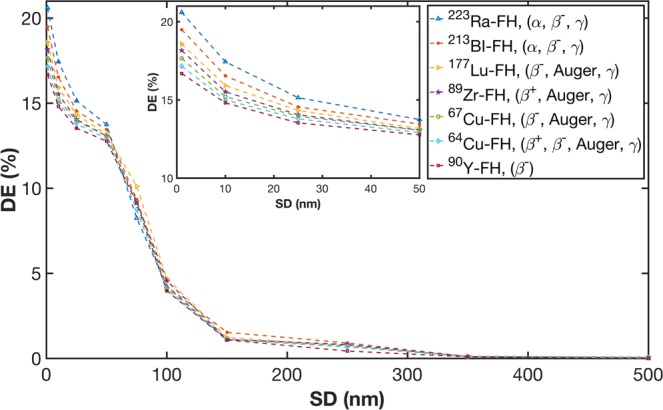
Dose enhancement (DE) for therapeutic and imaging isotopes labeled to Feraheme (FH) as function of nanoparticle separation distance (SD). The inset shows a zoom-in of the region for SDs up to 50 nm.

The dose enhancement percentage (DE) as a function of maximum NP SD is presented in Fig. 5. For all the radio-FH sources, a DE of 15–20% is achieved at SD <20 nm, which corresponds to the range of electrons in the peak of the secondary energy spectra (cf. Fig. 3). The DE decreases sharply for SD >50 nm and becomes negligible after SD >300 nm. The highest DE is achieved with ^223^Ra-FH. This is primarily due to the high LET of the emitted alphas (with maximum energy, is ≈5.9 MeV). In addition, ^223^Ra-FH has the highest total number of disintegrations (≈1.4 × 10^9^) for 1 kBq activity compared to the other radio-FH sources. Consequently, this results in the highest number of particle hits (≈2.8 × 10^9^) within the NPs (cf. Fig. 2). An additional contribution to DE arises from enhanced electron-impact ionization of NPs due to the β^−^ emission from ^223^Ra (4% weighted probability, with average energy 370 keV). Compared to water without NPs, the electron-ionization cross-section increases by approximately 15%. The emitted gammas (with 2% weighted probability and average energy ≈262 keV) have a negligible impact on nanoscale DE due to their mean attenuation length, ≈6.6 cm. ^213^Bi-FH, with similar spectral characteristics to ^223^Ra-FH, results in a similar DE. These results also predict the lowest DE for ^90^Y-FH, which is attributable to the relatively high range of the emitted betas and low yield of secondary electrons (cf. Fig. 3). It is noteworthy that the isotopes ^89^Zr and ^64^Cu which are generally used for nuclear medicine imaging also produce non-negligible DE in the presence of nanoparticles^41^.

### Radial dose distribution

**Figure 6:**
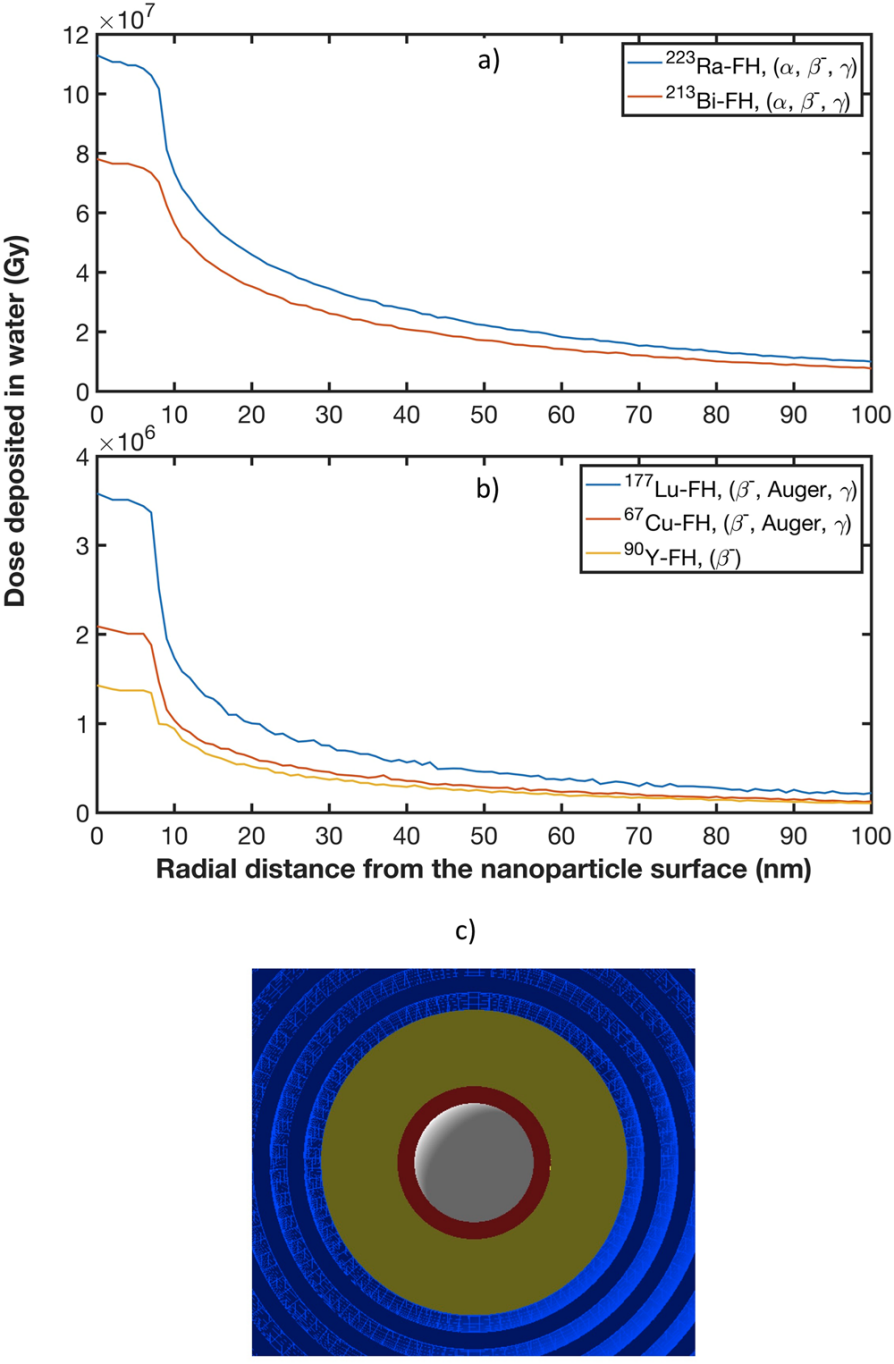
Radial doses are calculated for the following therapeutic radioisotopes: (**b**) ^213^Bi and ^223^Ra; and (**b**) ^90^Y, ^177^Lu and ^67^Cu (note the different dose scales in the two plots). (**c**) Cross section of the GATE simulation geometry set-up for radial dose distribution around a single radio-FH nanoparticle (white, red and yellow represent the SPIO core, radiolabeled layer and polymer coating, respectively): a detector comprising concentric spherical shells (light and dark blue) with equidistant intervals of 1 nm is centered on a single radio-FH nanoparticle and extends from its surface (at a radius of 8.5 nm) to a radius of 100 nm.

Figure 6(a,b) shows the radial dose distribution in water around a single radio-FH NP as a function of the radial distance from the NP surface. The particle emission from ^213^Bi, ^223^Ra, ^90^Y, ^177^Lu and ^67^Cu therapeutic radioisotopes results in significant energy deposition localized to within 10 nm of the NP surface, with doses on the order of 10^7^ Gy for ^213^Bi and ^223^Ra and 10^6^ Gy for the other isotopes. This extremely high energy deposition in an extremely small volume is relatively uncommon in external beam photon radiotherapy, since the incident radiation is considerably more sparsely ionizing^10^. In contrast, such dense dose distribution are more commonly observed in proton and heavy ion therapy (using high linear energy transfer (LET) particles)^42^ which has similar radiation characteristics to the radioisotopes used in this study (e.g. *α* particles and Auger electrons). These results also demonstrate that the alpha emitters ^213^Bi and ^223^Ra produce the highest localized energy deposition due to the densely ionizing property of alpha particles. For the beta emitting isotopes, our results corroborate those of a previous study^24^ where the emission of Auger electrons from ^125^I from the surface of a single gold nanoparticle (GNP) resulted in a localized dose (peaking at 2.50 ×10^9^ Gy) within 20 nm of the GNP.

## Discussion

Radiotherapy treatment is typically delivered with a radiation beam generated by a linear accelerator such that the x-ray photons have sufficient energy to reach a tumor buried deep inside the body. However, the MeV photon energies in such beams are incompatible with nanoparticle radio-enhancement, which requires energies several orders of magnitude lower to gain leverage from the photoelectric effect’s sensitive dependence on *Z*. This has been expounded through several simulation studies comparing radio-enhancement with MeV and keV x-ray beams for a range of different high-*Z* nanoparticles^5^. Simulation studies have further revealed, however, that a steep nanoscale dose gradient can arise in the immediate vicinity of a nanoparticle when irradiated by a clinical radiotherapy beam because such beams also contain keV photons as well as contaminant electrons and the relative contribution of this low-energy component increases with passage through tissue^42^. This partly explains why some evidence for nanoparticle radio-enhancement is observed in *in vitro* and *in vivo* studies using clinical MeV beams^5^. Needless to say, biological processes also play an important role, particularly as nanoparticles are known to be uptaken into the cytosol and organelles, where the ensuing radiobiological outcomes are more complex and less well known than in the nucleus where DNA is the primary target^43^. Nanoparticle radio-enhancement effects are unequivocally more dramatic for keV x-ray beams, with increases in median survival and/or reduction in tumor volume observed in *in vivo* studies^5^. This presents a challenge for clinical translation of nanoparticle radio-enhancement, however, as conventional keV beams are generally only used to treat superficial tumors and new treatment strategies need to be developed to treat deeply positioned tumors^44^. Furthermore, although the development of radionuclide therapy (RNT) provides a growing set of data on radiobiologic effects, the information on normal tissue toxicity in RNT is indeed still more limited than that from external beam radiotherapy (EBRT) and appears to be more variable^45–47^. In general in both RNT and EBRT, the dose-rate at which an administered dose is delivered is one of the most factors in both tumor control probability (TCP) and normal tissue complication probability (NTCP) since for both cancerous and healthy tissues the DNA repair rate competes with the radiation damage rate^48,49^. In addition, it is expected that RNT generally has a smaller TCP/NTCP ratio compared to EBRT due its low dose-rate in delivering a cumulative dose^45,46^. Therefore, dose enhancement by radiolabeled nanoparticles (radio-NPs) mainly enhances the cumulative dose (target organ cumulative uptake of radio-NPs) and not instantaneous dose (i.e. dose to the patient’s bloodstream). Thus, the radiation damage from radio-NP to normal tissue through the patient’s bloodstream depends on the targeting mechanism and can vary depending on the pharmacokinetics of the radio-NPs. Note, however, that the tolerance of normal tissues often appears to be greater in RNT than in EBRT^50–53^.

Furthermore, our study suggests that the highly localized energy deposition in the vicinity (<10 nm) of a radio-FH NP may potentially be very significant. The radial dose for each radio-FH NP exhibits a high degree of localized dose at a radial distance of up to 10 nm. This is mainly due to the high number of interactions of the primary radiation with NPs, producing numerous low energy, short-range secondary electrons, which in turn interact with the NPs due to their proximity. For a similar dose of X-rays (very sparsely ionizing radiation), densely ionizing charge particles (e.g. Auger electrons and *α* particles) are known to be more lethal and effective in killing cells and thus exhibit a higher Relative Biological Effectiveness (RBE)^24,54,55^. The RBE quantity is defined as the ratio of dose required to cause the same level of cell killing by sparsely ionizing radiation (i.e. X-rays) relative to more densely ionizing radiation (e.g. *α* particles)^56^.

This study opens up several possible avenues for further investigation into the radiobiological effectiveness of enhanced localized damage from radiolabeled NPs. The highly localized dose at radial distances within 10 nm from the surface of a single radio-FH NP, as well as the peak dose observed in NP clusters with SD up to 1 nm, demonstrate the dominance of short-range effects. This highlights the importance of sub-cellular uptake and localization. Upon forming a micro-cluster, radiolabeled nanoparticles that enter the cell nucleus can enhance DNA damage (relative to radiation damage without nanoparticles present) and thus trigger damage-signaling pathways^24^. Such nano-enhanced DNA damage will be most effective for nanoparticles labeled with radioisotopes emitting alpha particles, whose densely ionizing characteristics lead to a greater propensity for double-strand breaks across DNA’s ≈2 nm diameter helix. However, nanoparticle-enhanced radiation damage may also ensue even if a nanoparticle cluster is not uptaken by the nucleus, but rather by other radiosensitive organelles, such as mitochondria^43,57^. In addition, the exposure of nanoparticles to a biological environment can cause nanoparticle aggregation via surface destabilization or protein-protein interactions^58–61^. For this reason, it is necessary to consider the effects of nanoparticle clustering on dose-enhancement. As our results indicate, a potentially important effect is nonlinear amplification of dose-enhancement due to a cascading release of electrons in the nanometric spaces between nanoparticles^20^.

The proof-of-principle results of this *in silico* study will be used to guide and inform future *in vivo* experimental studies to test radio-FH in a lymph node metastases mouse model. Previous *in vivo* studies found^62^ that FH is uptaken by monocytes and trafficked to lymph nodes, where tumor cells first spread to. Thus, FH labeled with a radiotherapeutic isotope may offer a targeted and highly localized treatment strategy for lymph node metastases that could be further improved by nanoparticle radio-enhancement.

As a concept, nanoparticle radio-enhancement was originally conceived and developed in the context of conventional external beam radiotherapy. Clinically, this is categorized under the discipline of radiation oncology. On the other hand, in the separate clinical discipline of nuclear medicine, radiation is delivered to a tumor internally rather than externally and more importantly, the energies of radioisotopes use clinically are all in a range that can maximize nanoparticle radio-enhancement effects. In our study, we considered all radioisotopes that are in current clinical use. For example, ^223^Ra is used to treat bone metastases^63^, while ^90^Y is used to treat liver cancer^64^. We found that for all of these isotopes, radiation dose can be enhanced by up to 20% by SPIO nanoparticles with a concentration [Fe] = 0.1 mM that is comparable to that used clinically for diagnostic magnetic resonance imaging. This is important because many previous studies that showed radio-enhancement used clinically unfeasible nanoparticle dosage^5^.

Furthermore, FH or in general SPIO NPs can be radiolabeled with isotopes using a novel chelate-free technique^23^ in which the radioisotopes bind directly to the surface of the SPIO core. One of the advantages of this technique is that the loading capacity (the number of radiometals per nanoparticle) can be optimized by using a different molar ratio of radiometals to NPs. For example, for labeling 92.5 MBq of ^89^Zr (m_Zr_ ≈ 0.00625 nmoles, N_atoms_ ≈3.75 × 10^13^) with 1 mg of FH NPs (m_Fe_ ≈17.9 µM, N_Fe_ atoms ≈107 × 10^17^, NFH ≈184 × 10^13^), the ratio of ^89^Zr atoms to FH NPs is ≈1:50. In addition, the large surface area to volume ratio of FH NPs suggests that a single FH NP can be loaded with a high number of radiometal atoms^65^. Therefore the loading capacity can be customized for dose enhancement optimization; while the absorbed dose increases linearly with initial activity, the DE increases nonlinearly with dose. Moreover, for future clinical studies, the loading capacity can be customized according to target geometry to not only enhance dose but also to achieve optimal TCP and NTCP. For example for targeted radio-FH therapy of small lesions (e.g. micro-metastases) the FH NPs can be radiolabeled with a high specific activity of an alpha emitting isotope (i.e. emitting short range, high LET alpha particles) to achieve a highly localized dose to the micro-metastases while minimizing collateral damage to surrounding healthy tissues. In the case of localized radio-FH therapy of a solid tumor where radio-FH can be directly injected in the tumor site, the FH NPs can be radiolabeled with both a long range beta emitter (for sufficient dose spatial coverage) and a short range alpha emitter (to enhance the biological effective dose) to optimize therapeutic efficacy.

Out of all radionuclides, the radiometals are growing increasingly popular for theranostic applications^40,66^. Amongst the metal radioisotopes, copper isotopes (e.g. ^67^Cu and ^64^Cu) are excellent candidates for nano-theranostic applications due to the large variety of clinically relevant half-lives available (i.e. 0.16–62 h) and their clinically favourable emission characteristics (*β*^−^, *β*^+^, or EC), which are suitable for both imaging and therapy^67,68^, as well as their compatibility for chelate-free labeling onto SPIO-core nanoparticles^20^. In addition, recent clinical studies^69–71^ show great promise for ^177^Lu and ^213^Bi targeted radionuclide therapy. Therefore, radiolabeled SPIO nanoparticles such as radio-FH can offer a novel and effective theranostic platform in cancer medicine.

In conclusion, this study has demonstrated, for the first time, a proof-of-principle of nanoparticle radio-enhancement for internal radionuclide therapy using clinically relevant isotopes and SPIO-core nanoparticles, thus paving the way for clinical translation. Our results have revealed that in addition to the favorable spectral characteristics of the isotopes and their proximity to the nanoparticle, nanoparticle clustering results in a collective effect that amplifies nanoscale radiation damage effects by electron-mediated inter-nanoparticle interactions: electrons released from a nanoparticle in a cluster can interact with neighboring nanoparticles, thereby triggering a cascade effect that can lead to nonlinear amplification of local energy deposition. Therefore, optimal radio-enhancement is achieved when the inter-nanoparticle distance is less than the mean range of the secondary electrons. For the radioisotopes in this study, this corresponds to inter-nanoparticle distances <50 nm, with the strongest effects observed within 20 nm.

Our study used the condensed history approach in Geant4 to simulate particle transport. This approximation is likely to affect the accuracy of energy deposition on nano-metric scales. Track structure simulations using Geant4-DNA are better suited to nano-dosimetry simulations, but interaction cross-sections are only available for liquid water, so nanoparticle dose enhancement cannot be simulated. However, new Geant4-DNA track structure models are being developed for simulating dose enhancement by gold nanoparticles^72^ and it is anticipated these models will be extended to other high-Z materials.

## Methods

Monte Carlo (MC) particle transport modelling was carried out using GEANT4.10.3^73^ to simulate particle emissions from radioactive decay (see Supplementary Section S1.1) and using GATE version 8^74^ to calculate the energy deposition that results from particle interactions.

### Particle emission simulations

A GEANT4 model for simulating radioactive decay was developed by utilizing the radioactive decay hadronic package to simulate the radioactive decay processes for ^213^Bi, ^223^Ra, ^90^Y, ^177^Lu, ^67^Cu, ^64^Cu and ^89^Zr isotopes and record the radiation associated with the decay (e.g. *β*^−^, *β*^+^ and *γ* and *α*-emission) and the energy spectra of each type of radiation. All simulations were performed using GEANT4.10.3^73^. For each GEANT4 simulation 10^6^ decays of each unstable nuclei was simulated in an otherwise empty geometry. Also, any subsequent unstable daughter nuclei were allowed to further decay. The energy spectrum of decay particles was recorded separately for each radiation type (e.g. *α*, *β*^−^and *γ*) into different histograms. In addition, for ^64^Cu, the GEANT4 radioactive decay model was modified to obtain the energy spectrum of *β*^−^and *β*^+^ separately. The computed spectra for all isotopes were used as the source for the simulations in GATE. Further details and additional results are presented in Supplementary Section S1.2.

### Energy deposition simulations

Energy deposition simulations were performed using GATE version 8 which in turn uses GEANT4.10.3^73,74^. The low-energy electromagnetic physics package^75^ of GEANT4, which describes electron, photon, and light ion interactions over an energy range of 200 eV– GeV, was used. For the ionization process, the step size was limited to prevent a decrease of the stopping range by more than 10%, until the range of the particle becomes less than 100 nm. Additionally, geometrical step limitation was set to the *UseSafety* option for all the simulations to improve accuracy. Dose was recorded for the water phantom only (energy deposition to the nanoparticles was not recorded as self-absorption does not have a direct impact on dose enhancement).

### Geometry modeling

**Figure 7:**
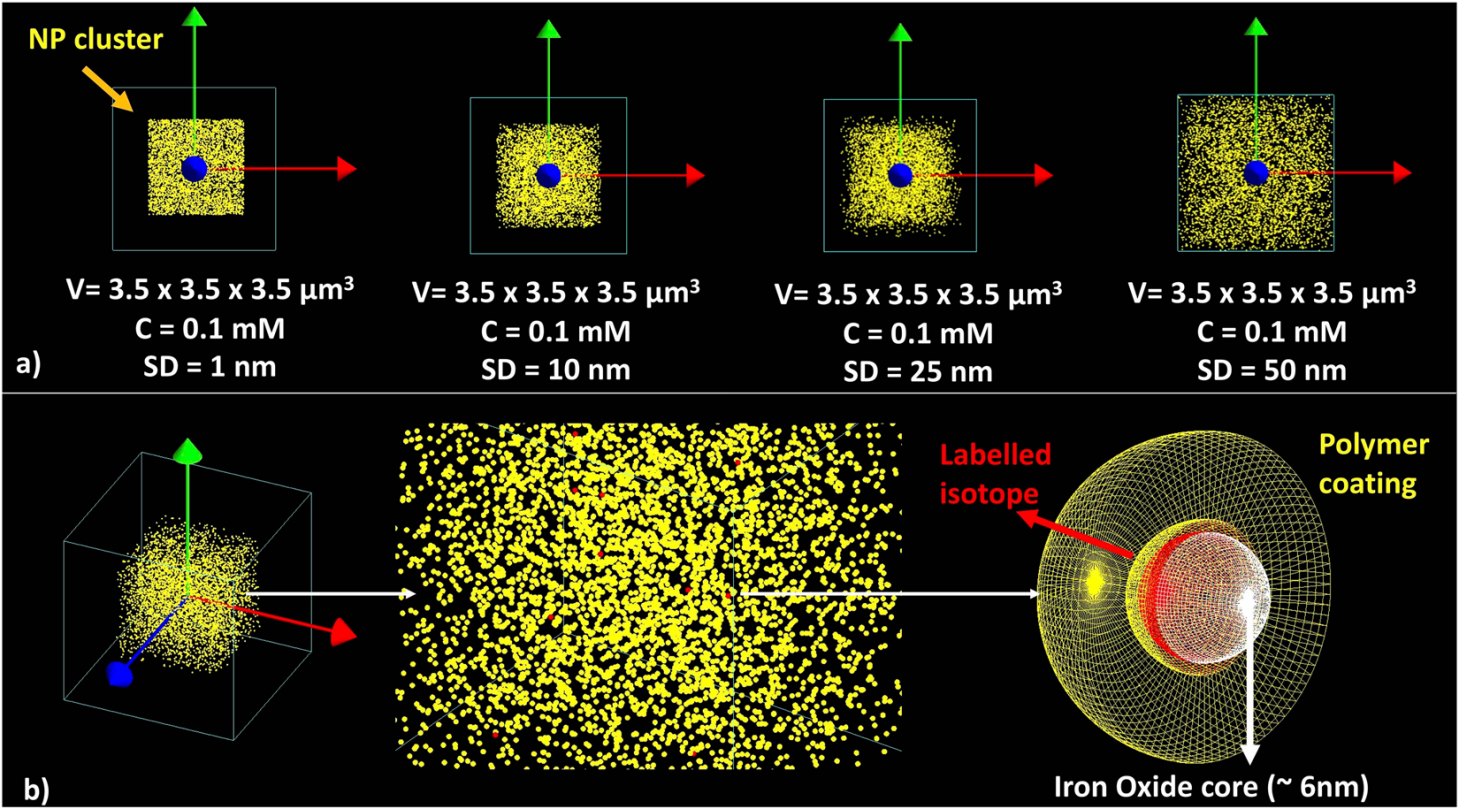
Geometry set-up in the GATE simulations: (**a**) A cluster of 500 spherical non-overlapping nanoparticles (NPs) were randomly distributed in a cubic volume of water (*V* ≈43 *μ*m^3^, corresponding to a concentration [Fe] = 0.1 mM) and the maximum NP separation distance (SD) was varied while keeping the volume and concentration the same; (**b**) 10 of the NPs were modelled as being radiolabeled, with the radioisotope uniformly distributed within a 1 nm wide ring around the iron oxide core.

A cluster of 500 spherical non-overlapping nanoparticles (NPs) was placed at the center of a ≈43 *μ*m^3^ water phantom (a 3.5 × 3.5 × 3.5 *μ*m^3^ box) to mimic NP dispersion in water with a concentration ≈0.1 mM Fe. The maximum separation distance (SD) was varied from 0 to 1, 10, 25, and 50 nm for each separate simulation (cf. Fig. 7(a)). Dose enhancement was also calculated by considering larger SDs (up to 500 nm) and by increasing the water phantom to 100 × 100 × 100 *μ*m^3^. Each NP was modeled as sphere with an SPIO core (diameter 6 nm) coated with polyglucose sorbitol carboxymethylether to mimic the core-shell geometry of an FDA-approved NP, Feraheme® (FH), which can be successfully radiolabeled with a range of isotopes using a chelate-free technique that binds the radioisotope directly to the SPIO core^23^ (cf. Fig. 7(b)). The overall FH NP (including the coating layer, with thickness of 5.5 nm) is ≈17 nm and the material for the core and the coating layer was set according to the stoichiometric formula: Fe_5874_ O_8752_:C_11719_ H_18682_ O_9933_ Na_414_. NPs were randomly distributed in the water phantom using a MATLAB code to generate cartesian coordinates of non-overlapping locations. The coordinates were input into GATE and NPs were distributed in the water phantom using the ‘genericRepeater’ algorithm. The NP volume overlap was checked again using the ‘overlap check option function’ in GATE. For each simulation, ten random NPs were selected and labeled with either ^213^Bi, ^223^Ra, ^90^Y, ^177^Lu, ^67^Cu, ^64^Cu or ^89^Zr source isotopes. A 1 nm layer of each radioisotopes was placed around the FH core to mimic radio-FH (cf. Fig. 7(b)).

For each simulation, the initial isotope activity *A*_0_ was set at 1 kBq. For ^90^Y, ^177^Lu, ^67^Cu, ^64^Cu and ^89^Zr isotopes, the total number of disintegrations was calculated using Eq. (1)

1 $$\tilde{A}={\int }_{0}^{\infty }{A}_{0}{e}^{-\lambda t}dt=\frac{{A}_{0}}{\lambda }$$

where $\tilde{A}$ and *λ* are the cumulative activity (i.e. the total number of disintegrations) and decay constant, respectively. As both ^213^Bi and ^223^Ra isotopes have decay chains with multiple daughter nuclei (with different branching ratios), the cumulative activity for these isotopes was calculated by integrating the Bateman Eq. (2)^76^

2 $${A}_{n}(t)=\frac{{A}_{1}(0)}{{\lambda }_{n}}\mathop{\sum }\limits_{i=1}^{n}{\lambda }_{i}{\alpha }_{i}\exp [-{\lambda }_{i}t]$$

where *A*_1_(0) is the initial number of nuclei in nucleus number 1 in the decay series and λ_*i*_ are the generalized decay constants, with

3 $${\alpha }_{i}=\mathop{\prod }\limits_{\begin{array}{c}j=1\\ j\ne i\end{array}}^{n}\frac{{\lambda }_{i}}{({\lambda }_{j}-{\lambda }_{i})}$$

A summary of these calculations is provided in Supplementary Table S1. For each simulation, three different GATE actors (dose, fluence and spectrum) were attached to the water phantom and the NPs to calculate two-dimensional (2D) histograms of energy deposition (E_dep_) within the water phantom, three-dimensional (3D) energy deposition histograms to calculate the energy volume histogram, 2D particle hits distributions within NPs, as well as Auger electron and photon spectra produced within the water phantom. Absorbed doses with and without NPs (i.e. replacing the NP material with water) were also computed and dose enhancement (DE) percentage values were calculated using Eq. (4):

4 $$DE( \% )=(DER\mbox{--}1)\times 100$$

where *DER* (i.e. dose in water with NPs/dose in water without NPs) is the dose enhancement ratio.

### Radial dose distribution

The radial distribution of dose in water was calculated around a single radio-FH NP by constructing a detector comprising concentric spherical shells with equidistant intervals of 1 nm from the NP surface up to 100 nm (Fig. 6c). The radio-FH NP was placed at the center of a 1 *μ*m^3^ water phantom box. The radial dose was obtained for each of the isotopes studies: ^213^Bi, ^223^Ra, ^90^Y, ^177^Lu and ^67^Cu.

All simulations were performed using the Geant4 (version 10.3) Livermore condensed history low-energy physics model, which has been shown to provide the best overall performance among other models (i.e. Penelope) for nanoscale electron transport^77–80^. The tracking and production range cuts were set to 10 eV and the maximum step size was set to 1 nm while the mean excitation energy of water and Fe were 75 eV and 286 eV respectively^81^. Secondary electrons below these thresholds were not simulated in this study. Atomic de-excitation including fluorescence, Auger production, and particle induced x-ray emission (PIXE) were activated. For the ionization process, the step size was limited (i.e. 0.1) to limit the stopping range decrease to 10% (which translates to a value of 0.1), until the particle range falls below 100 nm (which translates to a value of 0.1 mm). Additionally, geometrical step limitation was set to the *UseSafety* option for all the simulations to improve accuracy. The *UseSafety* option activates an additional restriction to ensure that a minimum number of steps are performed in any geometrical volume, even in low-density media or very thin layers. This additional restriction is computed at the start of each new track and recomputed only when entering a new volume^82^. Although Geant4 offers the Geant4-DNA low-energy physics models for electron transport at the nanoscale^72,83,84^, these models are only available for liquid water and thus could not be used in this study.

## Supplementary information

Supplementary Information
