# Supplementary material for: Radio-enhancement effects by radiolabeled nanoparticles
Source: Sci Rep. 2019 Oct 4;9:14346. doi: 10.1038/s41598-019-50861-2 (PMC6778074; doi:10.1038/s41598-019-50861-2)
Supplement: Supplementary file 1 — Supplementary Information [file 41598_2019_50861_MOESM1_ESM.pdf]

Radio-enhancement effects by radiolabeled nanoparticles

Yaser Hadi Gholami, Richard Maschmeyer, Zdenka Kuncic

1.1 Particle emission simulations.

A GEANT4 model for simulating radioactive decay was developed by utilizing the radioactive decay hadronic package to simulate the radioactive decay processes for ^{213}Bi, ^{223}Ra, ^{90}Y, ^{177}Lu, ^{67}Cu, ^{64}Cu and ^{89}Zr isotopes and record the generated secondary particles, radiation associated with the decay (e.g. β^-, β^+ and γ and α-emission) and the energy spectra of each type of radiation. All simulations were performed using GEANT4.10.3[1]. The radioactive decays simulation in GEANT4 is data driven (i.e. empirical or pre-calculated data) using a reprocessed 'The Evaluated Nuclear Structure Datafile (ENSDF)' library. The ENSDF library contains information such as half-life times, decay types, branching ratios, emission energies and transition types. The algorithm from this library samples any direct emission from a radioactive decay (i.e. α and β as well as neutrinos) resulting from nuclear transmutations. In addition, the sampling and production of nuclear and atomic deexcitation emission such as γ-emission, conversion electrons (CE), fluorescence emission (from the shell vacancies occurring from electron-capture decays) and Auger electron (AE) are handled by G4PhotonEvaporation and G4AtomicDeexcitation classes which are using separate set of data libraries.

For each GEANT4 simulation 10^6 decays of the above unstable nuclei was simulated in an otherwise empty geometry. Also, any subsequent unstable daughter nuclei were allowed for further decay. The energy spectrum of radiation and particles resulting from the decay of each isotope was recorded separately for each radiation type (e.g. α, β^-, γ) into different histograms. In addition, for ^{64}Cu isotope, the GEANT4 the radioactive decay model was further modified to obtain separate $\beta^-$and β^+ energy spectra. The maximum energies of the emitted $\beta^-$and β^+ particles (which are equivalent to Q, the total kinetic energy released per decay) were compared against the analytical calculations for validation. Equation 1 and 2 were used to calculate the maximum energies for β^+ and β^- particles:

$$Q_{\beta^-} = \left(\left[m_{^{64}_{29}Cu} - Zm_e\right] - \left[m_{^{64}_{30}Zn} - (Z+1)m_e\right] - m_e\right)C^2 \qquad (1)$$

$$Q_{\beta^+} = \left(\left[m_{^{64}_{29}Cu}\right] - \left[m_{^{64}_{28}Ni}\right] - 2m_e\right)C^2 \qquad (2)$$

Where Q_{β^-} and Q_{β^+} are the total kinetic energy released from β^+ and β^- decay processes, respectively. Z and m_e are also the atomic number and the rest mass of the electron, respectively. $m_{^{64}_{29}Cu}$, $m_{^{64}_{30}Zn}$, $m_{^{64}_{29}Cu}$ and $m_{^{64}_{28}Ni}$ are the $^{64}_{29}Cu$, $^{64}_{30}Zn$, $^{64}_{29}Cu$, $^{64}_{28}Ni$ nuclear masses, respectively.

1.2 The computed spectra for all isotopes

Radioactive decay was simulated differently depending on the type of radiation emitted by the radioisotope. For ^{223}Ra and ^{213}Bi isotopes, which are α-emitters, a He–nucleus was emitted from the parent nucleus resulting in daughter nuclei with two fewer protons and two fewer neutrons than the parent nuclei. The parent and daughter nuclei can subsequently undergo β^- decay, which produces a continuous energy spectrum. De-excitation by γ emission may also occur. Figure. 1(a-b) presents the energy spectra for α, β^- and γ emissions for the full decay scheme of ^{213}Bi and ^{223}Ra radioisotopes, respectively. The intensity is weighted relative to the total energy released per decay. The full decay scheme for each isotope is presented in Fig. 2 and 3, respectively.

^{177}Lu and ^{67}Cu are primarily β^- emitters, but also have a γ decay branch. The full decay spectra additionally include the emission of conversion electron (CE) and Auger electrons (AE). The energy spectra of β^-particles, γ, CEs and AEs from^{177}Lu and ^{67}Cu decays are shown in Fig. 4 (a-b). The full decay scheme for each isotope is presented in Fig. 5 and 6, respectively.

The full ^{64}Cu and ^{89}Zr decay scheme includes β^-, β^+ and γ emission, as well as AE emission. The ^{89}Zr β^+, ^{64}Cu β^- and β^+ energy spectra are shown in Fig. 7(a-b). The differences in the ^{64}Cu β^- and β^+ energy spectrum shapes at low energies is due to differences in the electron-nuclear and positron-nuclear Coulomb interactions, as a result of their opposite charges. The proportion of low-energy β^- particles exceeds that of low-energy positrons due to the nuclear Coulomb attraction and repulsion, respectively. Additionally, the analytical calculations (using Eq. (1) and (2)) for maximum energies of β^- and β^+ particles were 0.578 MeV and 0.653 MeV, respectively. These results are also consistent with the computed maximum energies and previously published data[2] (i.e. 0.577 MeV and 0.654 MeV, respectively). The full decay scheme for ^{64}Cu and ^{89}Zr presented in Fig. 8 and 9, respectively.

Figure 7(c) shows the emission spectrum for, ^{90}Y, which primarily decays via β^- decay (99.999%)[3]. However, the decay of ^{90}Y has a minor branch (0.011%) to the 0^+, first excited state of ^{90}Zr at 1.76 MeV, which is followed by a β^-/β^+ emission[4]. Single gamma emission is strictly forbidden for spin-zero to spin-zero transitions. Thus, a possible process that could occur is the transitions giving rise to transfer of radiation energy to an atomic electron in the orbital cloud by internal conversion. If the energy of the process is greater than $2m_ec$ (1.022 MeV, where m_e is the rest mass of the electron), transition can occur via electron-positron internal pair creation[3]. This internal pair production is generated by a rare electric monopole transition (E0) between the states 0+/0+ of ^{90}Zr. Since the β^+ emission yield from ^{90}Y decay is insignificant it was not included in the spectrum plot. The full decay scheme is presented in Fig. 10.

Figures

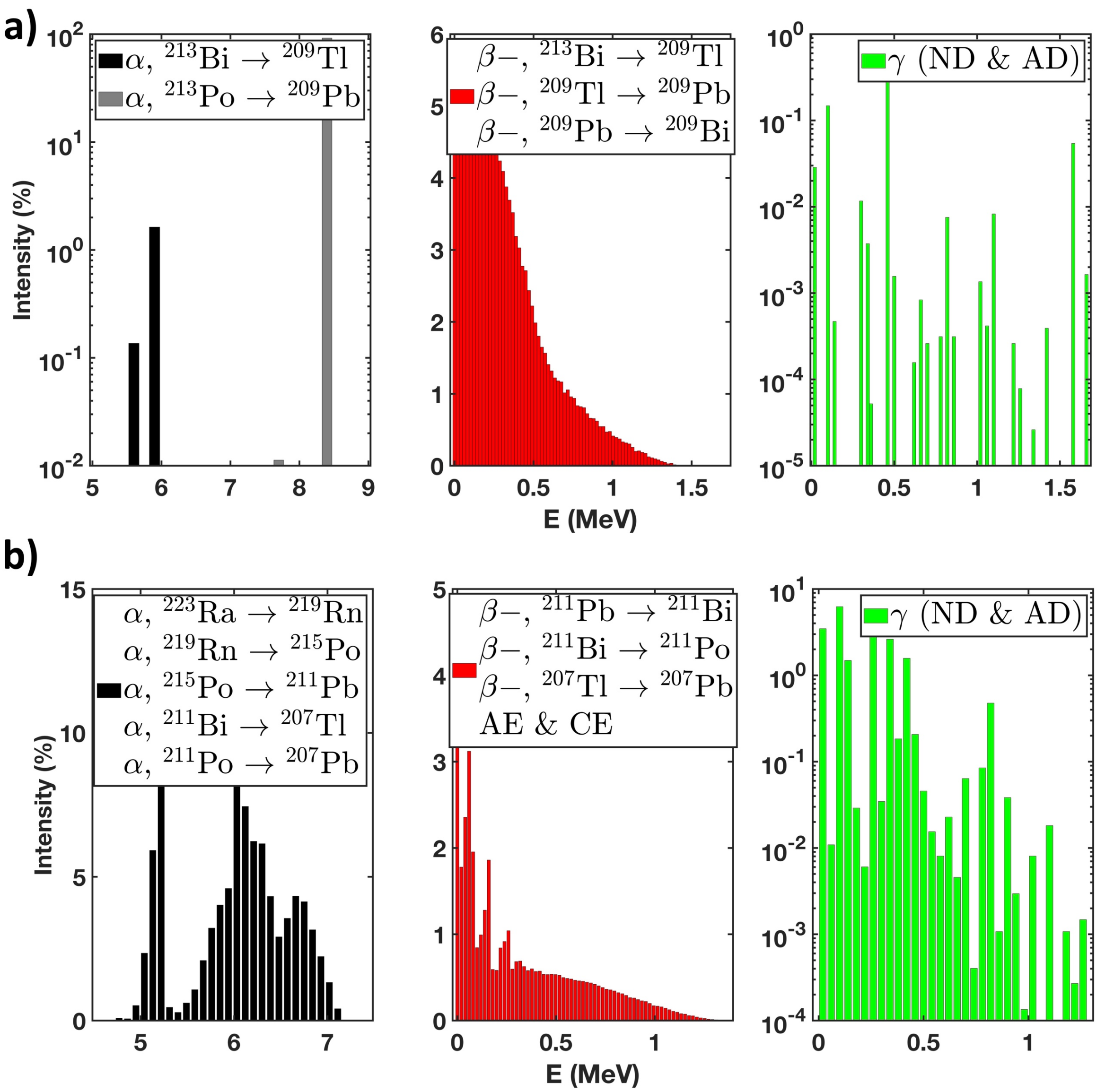

Figure 1. Full energy spectra for (a) ^{213}Bi and (b) ^{223}Ra isotopes. Abbreviation: nuclear-deexcitation (ND); atomic—deexcitation (AD); Auger electron (AE); conversion electron (CE).

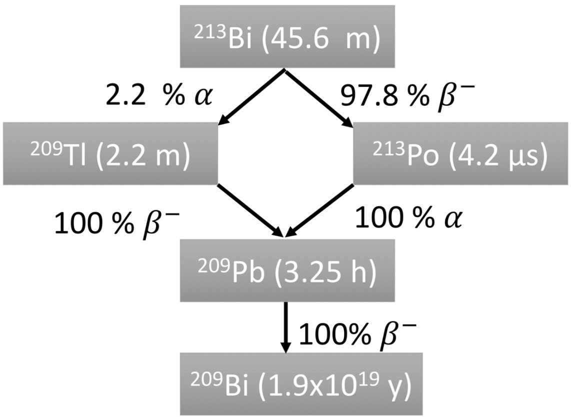

Figure 2. The decay scheme of ^{213}Bi radioisotope. Abbreviation: minute (m), second (s), hour (h) and year (y).

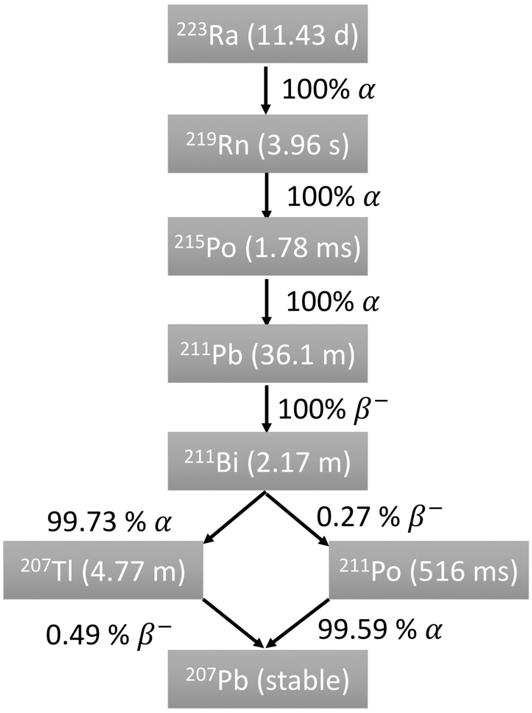

Figure 3. The decay scheme of ^{223}Ra radioisotope. Abbreviation: minute (m), second (s), hour (h) and year (y).

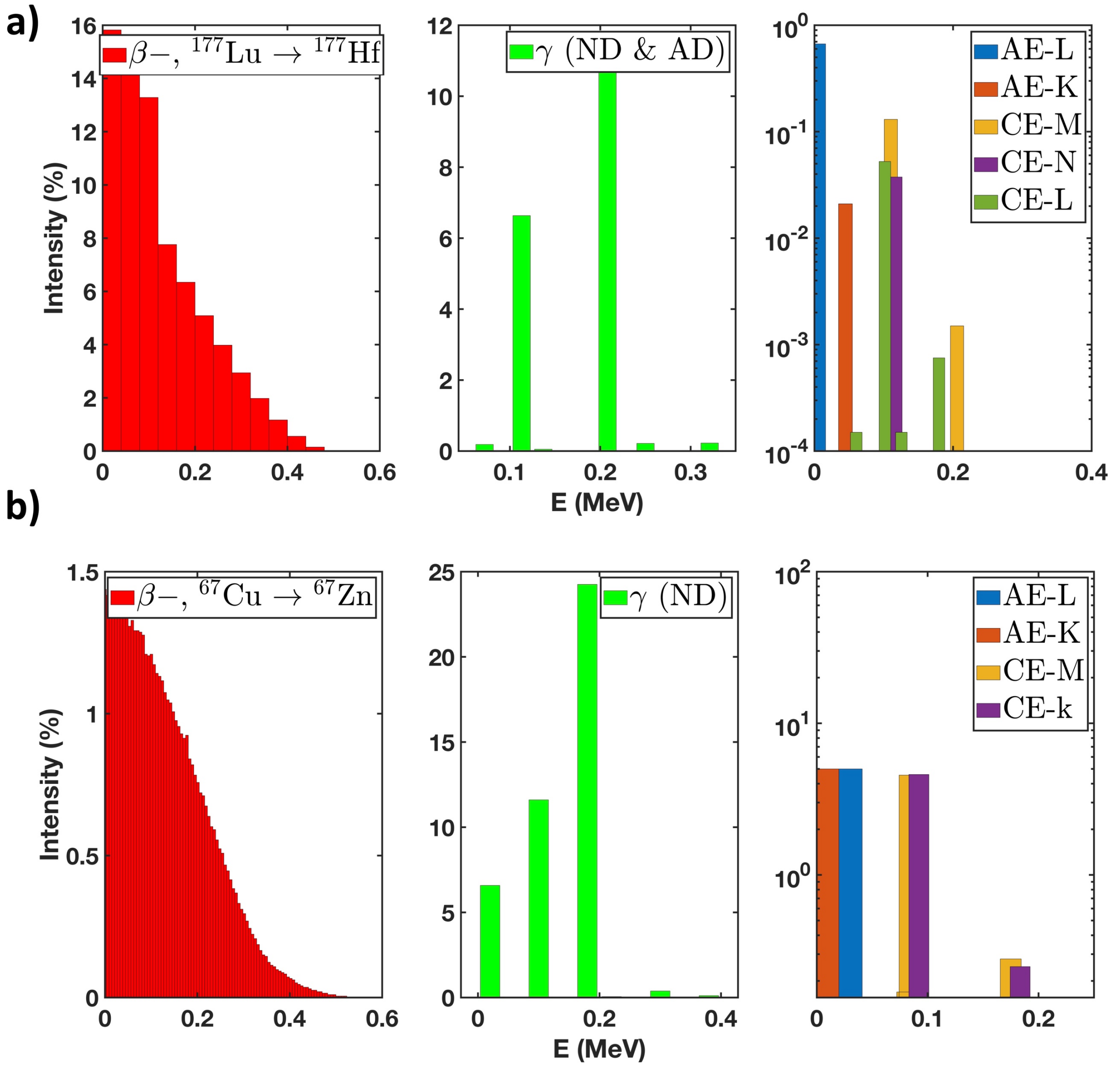

Figure 4. Full energy spectra for (a) ^{177}Lu and (b) ^{67}Cu isotopes. Abbreviation: nuclear-deexcitation (ND); atomic—deexcitation (AD); Auger electron (AE); conversion electron (CE); L, K and M are the atomic shells.

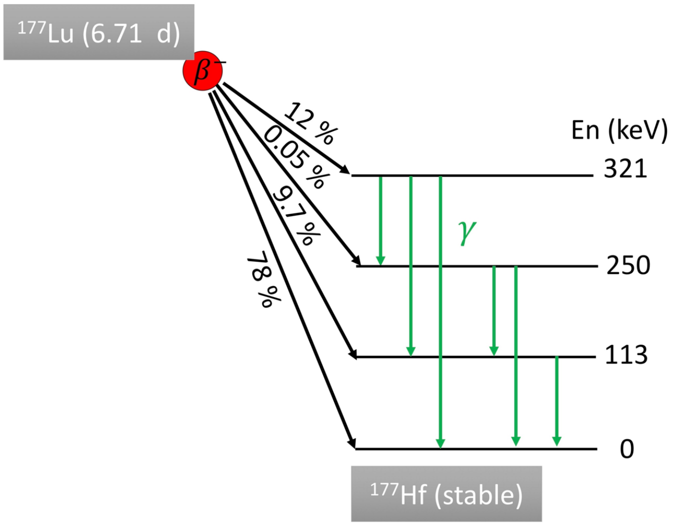

Figure 5. The decay scheme of ^{177}Lu radioisotope. Abbreviation: minute (m), second (s), hour (h), year (y) and energy level (En).

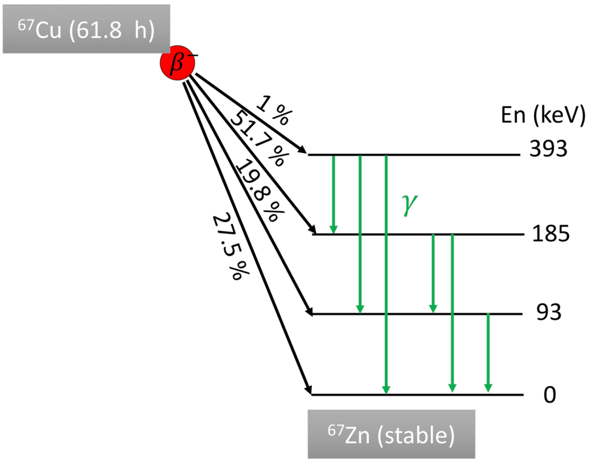

Figure 6. The decay scheme of ^{67}Cu radioisotope. Abbreviation: minute (m), second (s), hour (h), year (y) and energy level (En).

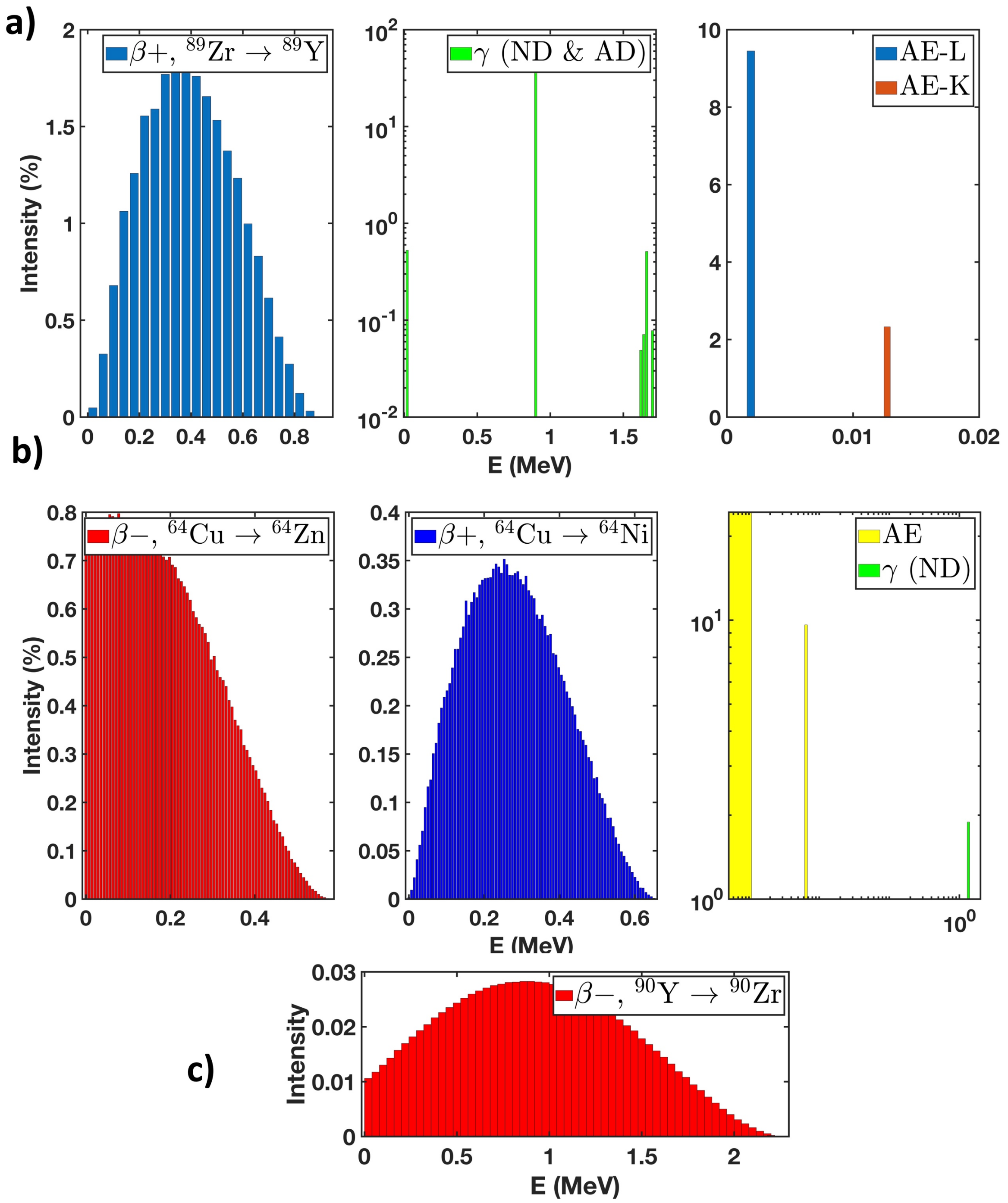

Figure 7. Full energy spectra for (a) ^{89}Zr, (b) ^{64}Cu and (c) ^{90}Y isotopes. Abbreviation: nuclear-deexcitation (ND); atomic—deexcitation (AD); Auger electron (AE); conversion electron (CE); L, K and M are the atomic shells.

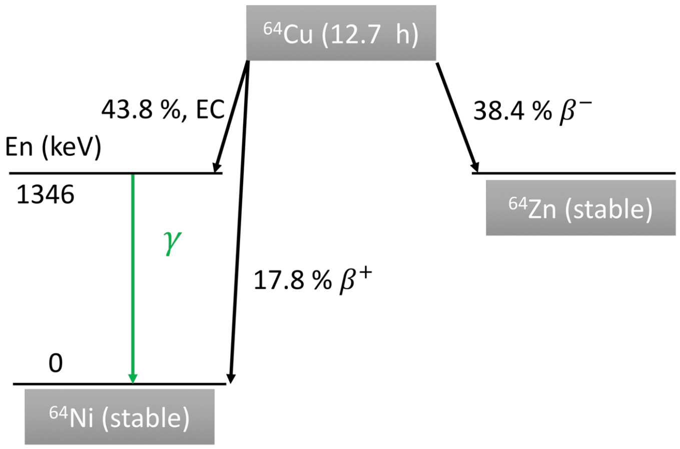

Figure 8. The decay scheme of ^{64}Cu. Abbreviation: hour (h) and energy level (En), electron capture (EC).

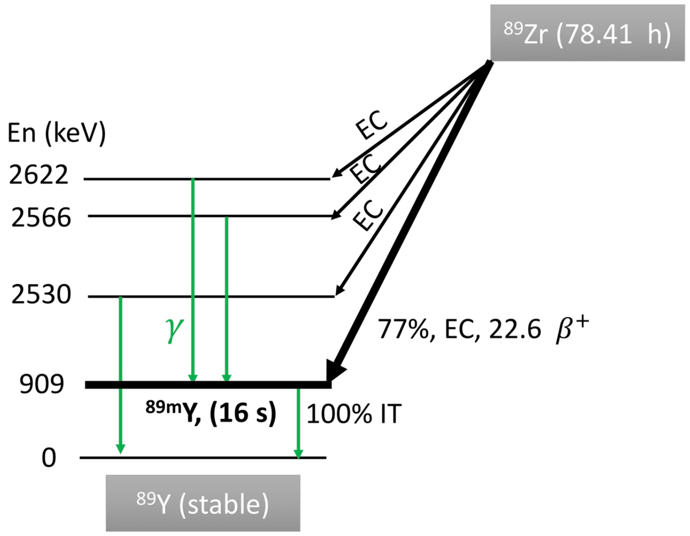

Figure 9. The decay scheme of ^{64}Cu radioisotope. Abbreviation: hour (h), energy level (En), electron capture (EC), isomeric transition (IT).

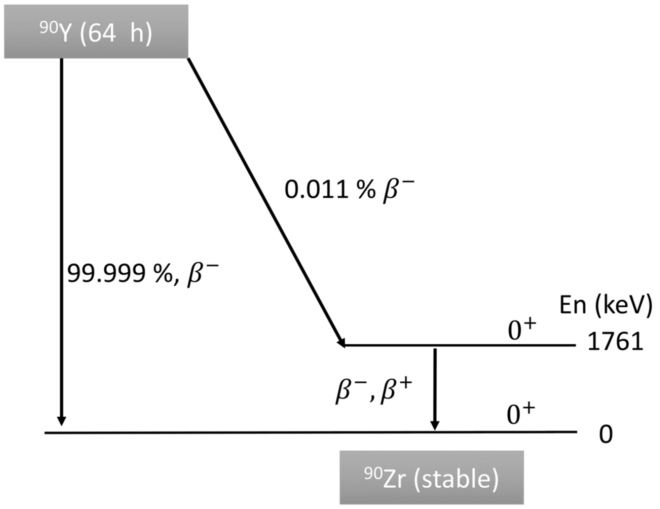

Figure 10. The decay scheme of ^{90}Y radioisotope. Note that β^-, β^+ particles emission is generated from Internal Pair production via 0^+- 0^+ transition of ^{90}Zr isotope. Abbreviation: energy level (En).

Tables

Table 1. Total number of disintegrations for 1 kBq activity with relative particle intensities. The intensity for each type of radiation is weighted relative to the total energy released per decay. Abbreviations: alpha particle, α; beta particle β^-; positron β^+, gamma γ, Auger electron and conversion electron, AE and CE, respectively.

**Isotopes**	**Total no of disintegrations**	**α (%)**	**β- (%)**	**β+ (%)**	**γ (%)**	**AE, CE (%)**
^{13}Bi	8.09E+06	93.50	5.77	0.00	0.73	0.00
^{223}Ra	1.43E+09	94.00	4.00	0.00	2.00	0.00
^{177}Lu	8.39E+08	0.00	74.05	0.00	18.45	7.50
^{67}Cu	3.21E+08	0.00	52.00	0.00	43.00	5.00
^{64}Cu	6.60E+07	0.00	38.50	17.60	35.90	8.00
^{90}Y	3.33E+08	0.00	100.00	0.00	0.00	0.00
^{89}Zr	4.07E+08	0.00	0.00	22.74	67.81	9.45

References

1. Agostinelli, S. *et al*. Geant4—a simulation toolkit. *Nucl. Instrum. Methods Phys. Res. Sect. Accel. Spectrometers Detect. Assoc. Equip.* **506**, 250–303 (2003).
2. Singh, B. Nuclear Data Sheets for A = 64. *Nucl. Data Sheets* **108**, 197–364 (2007).
3. Kossert, K. & Schrader, H. Activity standardization by liquid scintillation counting and half-life measurements of 90Y. *Appl. Radiat. Isot.* **60**, 741–749 (2004).
4. Selwyn, R. G., Nickles, R. J., Thomadsen, B. R., DeWerd, L. A. & Micka, J. A. A new internal pair production branching ratio of 90Y: the development of a non-destructive assay for 90Y and 90Sr. *Appl. Radiat. Isot. Data Instrum. Methods Use Agric. Ind. Med.* **65**, 318–327 (2007).
